# Supplementary material for: Feasibility of Dynamic Inhaled Gas MRI-Based Measurements Using Acceleration Combined with the Stretched Exponential Model
Source: Diagnostics (Basel). 2023 Jan 30;13(3):506. doi: 10.3390/diagnostics13030506 (PMC9914115; doi:10.3390/diagnostics13030506)
Supplement: Supplementary file 1 [file diagnostics-13-00506-s001.zip › diagnostics-2036686-supplementary.pdf]

Table S1. ^{3}He MRI-based Fractional-Ventilation Measurements

FGRE AF10	r	r_{sem}	r^A	r_{sem}^A	$r - r^A$	$r - r_{sem}$	$r_{sem} - r^A$	$r^A - r_{sem}^A$
**Rat 1**	0.22(.03)	0.21(.01)	0.22(.03)	0.21(.02)	4%	1%	5%	9%
**Rat 2**	0.23(.02)	0.21(.01)	0.23(.02)	0.21(.01)	4%	6%	4%	6%
**Rat 3**	0.21(.02)	0.19(.01)	0.21(.03)	0.19(.01)	4%	5%	4%	5%
**Rat 4**	0.21(.02)	0.19(.01)	0.21(.03)	0.19(.01)	4%	5%	4%	5%
**Rat 5**	0.21(.03)	0.18(.02)	0.21(.03)	0.18(.02)	5%	7%	6%	8%
**FGRE AF14**								
**Rat 1**	0.22(.03)	0.21(.01)	0.22(.03)	0.21(.04)	2%	1%	9%	11%
**Rat 2**	0.23(.02)	0.21(.01)	0.23(.03)	0.21(.03)	7%	6%	6%	6%
**Rat 3**	0.21(.02)	0.19(.01)	0.21(.03)	0.19(.03)	7%	6%	7%	6%
**Rat 4**	0.21(.02)	0.19(.01)	0.21(.03)	0.19(.03)	7%	6%	7%	6%
**Rat 5**	0.21(.03)	0.18(.02)	0.21(.03)	0.18(.02)	6%	7%	8%	7%
**XC AF10**								
**Rat 1**	0.22(.03)	0.21(.01)	0.22(.03)	0.21(.02)	4%	1%	5%	10%
**Rat 2**	0.23(.02)	0.21(.01)	0.23(.02)	0.21(.02)	6%	6%	4%	7%
**Rat 3**	0.21(.02)	0.19(.01)	0.21(.03)	0.19(.02)	4%	6%	4%	6%
**Rat 4**	0.21(.02)	0.19(.01)	0.21(.03)	0.19(.02)	4%	6%	4%	6%
**Rat 5**	0.21(.03)	0.18(.02)	0.21(.03)	0.18(.01)	3%	7%	5%	8%
**XC AF14**								
**Rat 1**	0.22(.03)	0.21(.01)	0.22(.03)	0.21(.02)	3%	1%	5%	10%
**Rat 2**	0.23(.02)	0.21(.01)	0.23(.02)	0.21(.01)	5%	6%	4%	7%
**Rat 3**	0.21(.02)	0.19(.01)	0.21(.02)	0.19(.01)	4%	6%	5%	7%
**Rat 4**	0.21(.02)	0.19(.01)	0.21(.02)	0.19(.01)	4%	6%	5%	7%
**Rat 5**	0.21(.03)	0.18(.02)	0.21(.03)	0.18(.02)	4%	7%	5%	8%

r = MRI mean fractional ventilation estimate obtained with the Deninger method using fully-sampled data; r_{sem} = MRI mean fractional ventilation estimate obtained with SEM using fully-sampled data; r^A= MRI mean fractional ventilation estimate obtained with the Deninger method using accelerated data; r_{sem}^A = MRI mean fractional ventilation estimate obtained with SEM using accelerated data; $r - r^A$/$r - r_{sem}$/$r_{sem} - r^A$/ $r^A - r_{sem}^A$/ = pixel-by-pixel deference between the fractional ventilation maps; SEM = stretched exponential model; AF=acceleration factor; FGRE=Fast Gradient Recall Echo; XC=x-Centric.

Table S2. ^{129}Xe MRI-based Fractional-Ventilation Measurements

**FGRE AF10**	r	r_{sem}	r^{A}	$r_{sem}{}^{A}$	r - r^{A}	r - r_{sem}	r_{sem} - r^{A}	r^{A} - $r_{sem}{}^{A}$
**Rat 1**	0.25(.02)	0.24(.02)	0.24(.02)	0.24(.02)	7%	7%	7%	8%
**Rat 2**	0.20(.03)	0.21(.03)	0.20(.02)	0.21(.02)	3%	9%	7%	16%
**Rat 3**	0.21(.04)	0.21(.04)	0.20(.03)	0.21(.03)	4%	9%	8%	14%
**Rat 4**	0.22(.02)	0.22(.02)	0.22(.02)	0.22(.02)	1%	3%	6%	13%
**Rat 5**	0.22(.02)	0.22(.02)	0.22(.02)	0.22(.02)	4%	1%	6%	11%
**Rat 6**	0.22(.02)	0.22(.01)	0.22(.02)	0.22(.02)	7%	8%	7%	12%
**Rat 7**	0.21(.03)	0.21(.02)	0.21(.02)	0.21(.02)	6%	1%	7%	11%
**FGRE AF14**								
**Rat 1**	0.25(.02)	0.24(.02)	0.24(.02)	0.24(.02)	13%	7%	10%	10%
**Rat 2**	0.20(.03)	0.21(.03)	0.20(.03)	0.21(.03)	6%	10%	9%	17%
**Rat 3**	0.21(.04)	0.21(.04)	0.20(.03)	0.20(.03)	7%	9%	9%	14%
**Rat 4**	0.22(.02)	0.22(.02)	0.21(.02)	0.22(.03)	7%	3%	9%	11%
**Rat 5**	0.22(.02)	0.22(.02)	0.22(.02)	0.22(.03)	1%	.04%	10%	12%
**Rat 6**	0.22(.02)	0.22(.01)	0.21(.02)	0.22(.02)	8%	6%	10%	13%
**Rat 7**	0.21(.03)	0.21(.02)	0.20(.03)	0.20(.03)	9%	10%	10%	14%
**XC AF10**								
**Rat 1**	0.25(.02)	0.24(.02)	0.24(.02)	0.24(.02)	6%	7%	7%	9%
**Rat 2**	0.20(.03)	0.21(.03)	0.20(.03)	0.21(.03)	5%	9%	8%	17%
**Rat 3**	0.21(.04)	0.21(.04)	0.20(.03)	0.20(.03)	4%	9%	6%	15%
**Rat 4**	0.22(.02)	0.22(.02)	0.21(.02)	0.22(.03)	8%	3%	8%	13%
**Rat 5**	0.22(.02)	0.22(.02)	0.22(.02)	0.22(.03)	5%	.03%	7%	13%
**Rat 6**	0.22(.02)	0.22(.01)	0.21(.02)	0.22(.02)	4%	6%	7%	12%
**Rat 7**	0.21(.03)	0.21(.02)	0.20(.03)	0.20(.03)	8%	10%	9%	13%
**XC AF14**								
**Rat 1**	0.25(.02)	0.24(.02)	0.24(.02)	0.24(.02)	7%	7%	7%	9%
**Rat 2**	0.20(.03)	0.21(.03)	0.20(.02)	0.20(.02)	4%	9%	9%	16%
**Rat 3**	0.21(.04)	0.21(.04)	0.20(.03)	0.20(.03)	5%	9%	8%	13%
**Rat 4**	0.22(.02)	0.22(.02)	0.21(.02)	0.22(.02)	7%	3%	7%	13%
**Rat 5**	0.22(.02)	0.22(.02)	0.21(.02)	0.21(.02)	.02%	.06%	7%	12%
**Rat 6**	0.22(.02)	0.22(.01)	0.21(.02)	0.21(.02)	5%	6%	8%	13%
**Rat 7**	0.21(.03)	0.21(.02)	0.20(.02)	0.20(.02)	7%	10%	9%	13%

r = MRI mean fractional ventilation estimate obtained with the Deninger method using fully-sampled data; r_{sem} = MRI mean fractional ventilation estimate obtained with SEM using fully-sampled data; r^{A}= MRI mean fractional ventilation estimate obtained with the Deninger method using accelerated data; $r_{sem}{}^{A}$ = MRI mean fractional ventilation estimate obtained with SEM using accelerated data; r - r^{A}/r - r_{sem}/r_{sem} - r^{A}/ r^{A} - $r_{sem}{}^{A}$/ = pixel-by-pixel deference between the fractional ventilation maps; SEM = stretched exponential model; AF=acceleration factor; FGRE=Fast Gradient Recall Echo; XC=x-Centric.

Table S3. ^{19}F MRI-based Fractional-Ventilation Measurements

**<u>FGRE AF10</u>**	r	r_{sem}	r^A	r_{sem}^A	$r - r^A$	$r - r_{sem}$	$r_{sem} - r^A$	$r^A - r_{sem}^A$
**Rat 1**	0.26(.04)	0.25(.09)	0.24(.02)	0.24(.10)	5%	13%	9%	11%
**Rat 2**	0.26(.05)	0.25(.09)	0.23(.03)	0.24(.06)	18%	16%	14%	15%
**Rat 3**	0.23(.05)	0.23(.08)	0.21(.04)	0.21(.06)	4%	16%	13%	18%
**Rat 4**	0.19(.07)	0.20(.09)	0.16(.05)	0.17(.06)	6%	19%	17%	87%
**Rat 5**	0.07(.05)	0.09(.07)	0.04(.02)	0.03(.02)	36%	15%	38%	50%
**Rat 6**	0.26(.04)	0.25(.09)	0.24(.02)	0.24(.10)	5%	13%	9%	11%
**<u>FGRE AF14</u>**								
**Rat 1**	0.26(.04)	0.25(.09)	0.24(.03)	0.24(.10)	10%	13%	10%	12%
**Rat 2**	0.26(.05)	0.25(.09)	0.22(.04)	0.24(.09)	21%	16%	16%	13%
**Rat 3**	0.23(.05)	0.23(.08)	0.20(.04)	0.21(.09)	8%	16%	18%	10%
**Rat 4**	0.19(.07)	0.20(.09)	0.16(.05)	0.19(.09)	10%	20%	15%	4%
**Rat 5**	0.07(.05)	0.09(.07)	0.04(.02)	0.02(.02)	35%	9%	20%	135%
**Rat 6**	0.26(.04)	0.25(.09)	0.24(.03)	0.24(.10)	10%	12%	10%	12%
**<u>XC AF10</u>**								
**Rat 1**	0.26(.04)	0.25(.09)	0.24(.02)	0.23(.08)	6%	13%	10%	12%
**Rat 2**	0.26(.05)	0.25(.09)	0.22(.04)	0.23(.05)	17%	15%	16%	11%
**Rat 3**	0.23(.05)	0.23(.08)	0.20(.04)	0.21(.07)	7%	16%	16%	17%
**Rat 4**	0.19(.07)	0.20(.09)	0.15(.05)	0.18(.09)	9%	19%	17%	11%
**Rat 5**	0.07(.05)	0.09(.07)	0.04(.03)	0.03(.03)	37%	15%	6%	206%
**Rat 6**	0.26(.04)	0.25(.09)	0.24(.02)	0.24(.08)	6%	13%	10%	11%
**<u>XC AF14</u>**								
**Rat 1**	0.26(.04)	0.25(.09)	0.24(.02)	0.24(.08)	7%	13%	11%	13%
**Rat 2**	0.26(.05)	0.25(.09)	0.22(.04)	0.23(.06)	20%	15%	17%	11%
**Rat 3**	0.23(.05)	0.23(.08)	0.20(.04)	0.21(.08)	6%	16%	16%	20%
**Rat 4**	0.19(.07)	0.20(.09)	0.15(.05)	0.18(.10)	5%	19%	18%	14%
**Rat 5**	0.07(.05)	0.09(.07)	0.04(.03)	0.03(.03)	35%	20%	.06%	133%
**Rat 6**	0.26(.04)	0.25(.09)	0.24(.02)	0.24(.08)	7%	13%	11%	13%

r = MRI mean fractional ventilation estimate obtained with the Deninger method using fully-sampled data; r_{sem} = MRI mean fractional ventilation estimate obtained with SEM using fully-sampled data; r^A= MRI mean fractional ventilation estimate obtained with the Deninger method using accelerated data; r_{sem}^A = MRI mean fractional ventilation estimate obtained with SEM using accelerated data; $r - r^A$/$r - r_{sem}$/$r_{sem} - r^A$/ $r^A - r_{sem}^A$/ = pixel-by-pixel deference between the fractional ventilation maps; SEM = stretched exponential model; AF=acceleration factor; FGRE=Fast Gradient Recall Echo; XC=x-Centric. The first two columns of Table show the r values obtained for AF=1 with SEM and Denninger method.

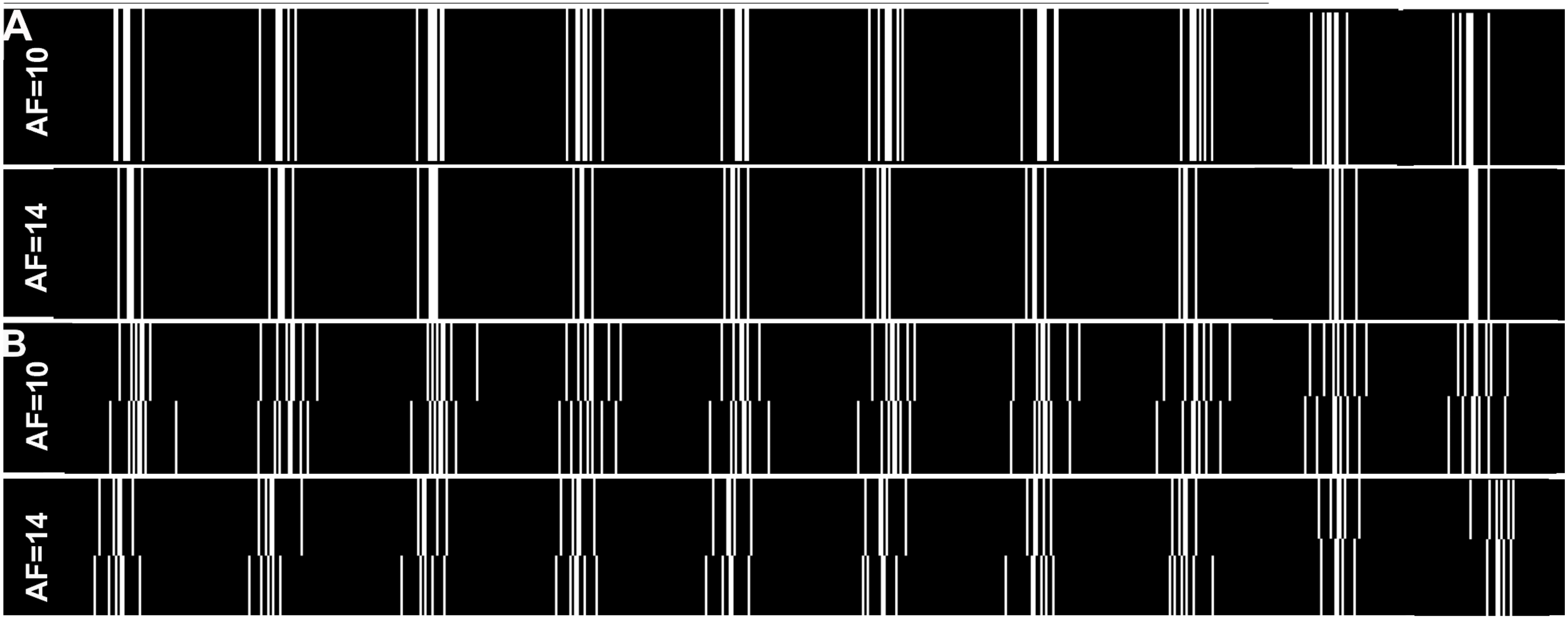

Figure S1. k-space under-sampling in the phase encoding direction (fully sampled in the x-direction) schemes, ensuring a variety of sparsity patterns for each wash-out ^{19}F image is depicted by A) AF=10 and AF=14 obtained for FGRE and B) AF=10 and AF=14 obtained for x-Centric that were retrospectively applied in wash-out direction. No undersampling in the x-direction was used for FGRE while 50% under-sampling (compensated by doubling the lines in the phase encoding direction) was employed for x-Centric.
